# The effect of the COVID-19 pandemic on disgust sensitivity in a sample of UK adults

**DOI:** 10.3389/fpubh.2022.1020850

**Published:** 2022-10-27

**Authors:** Peter Carr, Emily Breese, Christopher J. Heath, Rachel McMullan

**Affiliations:** ^1^School of Life, Health and Chemical Sciences, The Open University, Milton Keynes, United Kingdom; ^2^The Alan Turing Institute, London, United Kingdom

**Keywords:** disgust sensitivity, COVID-19, disease avoidance, behavior, pathogen disgust

## Abstract

The COVID-19 pandemic led to the introduction of a range of infection prevention and control (IPC) measures that resulted in dramatic changes in people's lives however these IPC measures are not practiced consistently across the population. One predictor of an individual's responses to the pandemic is disgust sensitivity. Understanding how disgust sensitivity varies within the population could help to inform design of public health messages to promote more uniform behavioral change during future pandemics. To understand the effect of the current COVID-19 pandemic on an individual's pathogen disgust sensitivity we have compared pathogen disgust sensitivity during the current COVID-19 pandemic to baseline pathogen disgust sensitivity, determined prior to the COVID-19 pandemic, in the same sample of UK adults. We find that the COVID-19 pandemic did not alter overall pathogen disgust sensitivity suggesting that disgust sensitivity is stable despite IPC measures, public health messaging, media coverage and other factors associated with the COVID-19 pandemic.

## Introduction

In March 2020 the World Health Organization (WHO) declared the outbreak of COVID-19 a global pandemic (1). As of 20^th^ December 2021 there were over 250 million confirmed cases of COVID-19 worldwide and over 5 million deaths (2). In the UK the government introduced social distancing and social isolation guidelines, restrictions on public gatherings and recommended a number of preventative health behaviors, including washing hands more frequently, in late March 2020 (3). These infection prevention and control (IPC) measures prompted many people to dramatically change their everyday lives in order to avoid contracting and spreading COVID-19. However, IPC measures are not practiced consistently. In the month after the UK government introduced social distancing measures the UK police force issued more than 9,000 fixed penalty fines (4) under new public health regulations (5) aimed at enforcing the lockdown suggesting that some people did not comply with guidelines. Conversely, a survey of Britons conducted by IPSO-MORI during the lockdown at the end of April 2020 suggested that some people would feel uncomfortable returning to “normal” activities such as visiting friends and family even once the lockdown restrictions were lifted (6).

What underlies these differences in behavior? Engagement with IPC measures is likely to be multi-factorial with an individual's engagement being influenced by a number of demographic and psychological factors such as functional fear, risk perception, socioeconomic status, disgust, engagement with social media, belief in conspiracy theories and moral values regarding the importance of caring for others. A number of studies have shown that pre-pandemic disgust sensitivity and proneness are important predictors of an individual's responses to the current COVID-19 global pandemic (7–10). Disgust sensitivity and proneness correlate with anxiety related to COVID-19 and predicts levels of concern about COVID-19 and efforts to comply with official recommendations (7–10). These studies are broadly in agreement with studies during previous pandemics (11), and disease outbreaks (12) which found disgust sensitivity to be a predictor of disease-related anxiety.

The emotion of disgust is the psychological mechanism for producing disease avoidance behaviors that protect us from infection by reducing our contact with pathogens and parasites (13). This pathogen avoidance theory of disgust (PAT) (13, 14) is supported by strong correlations between disgust elicitors and pathogen sources (15, 16). Sensitivity to disgust elicitors varies considerably amongst individuals (16) and one prediction derived from the pathogen-avoidance theory is that disgust sensitivity will be higher when the threat of infection is higher.

In support of this hypothesis Skolnick and Dzkoto (17) have demonstrated an association between disgust sensitivity and differing levels of national pathogen stress in Ghana and the USA however existing data do not fully support this association. Tybur et al. (18) comparing over 30 nations with differing levels of national parasite stress failed to find a correlation between disgust sensitivity and national rates of infectious diseases while a similar pattern of disgust sensitivity was found across nine different cultural regions using photo-based disgust stimuli (14). Overall, the findings of these studies do not consistently support the hypothesis that pathogen disgust sensitivity is correlated with vulnerability to infection therefore further research is warranted.

According to PAT, it could be hypothesized that disgust sensitivity should be higher during the COVID-19 pandemic in response to the increased real and perceived threat of infection globally. Recent studies have supported this hypothesis by demonstrating an impact of the COVID-19 pandemic on disgust sensitivity (19, 20). Using the Disgust Scale (16) to assess disgust sensitivity Stevenson et al. (20) reported overall higher levels of disgust sensitivity and higher scores for core disgust in a cohort of Australian university students during the first Australian lockdown in March/April 2020 when compared to previous cohorts of university students. Consistent with this, a study by Milkowska et al. (19) found that a cohort of women living in Poland assessed photographs depicting sources of infection as more disgusting during the COVID-19 pandemic when compared to a matched, pre-pandemic cohort. However, using questions adapted from the pathogen and moral disgust domains of the Three-Domain Disgust scale (21), the same study found a reduction in moral disgust during the pandemic when compared to the pre-pandemic cohort and no significant effect of COVID-19 on the pathogen disgust domain (19).

One limitation of these studies is their between-subjects design in which data was collected from different cohorts of individuals pre- and post-pandemic which does not allow disgust sensitivity in the same group of individuals to be compared pre- and post-pandemic. Thus, despite cohorts being matched, it remains possible that the observed differences in disgust sensitivity are a result of variation between individuals in the cohorts rather than as a result of increase threat of infection during the pandemic. Here we report a comparison of pathogen disgust sensitivity during the COVID-19 pandemic to baseline pathogen disgust sensitivity, determined in the same individuals prior to the COVID-19 pandemic, in order to better understand the effect of the current COVID-19 pandemic on pathogen disgust sensitivity.

## Materials and methods

### Survey instrument

A pathogen disgust survey comprised of 30 disgust elicitor statements derived from infectious disease transmission routes that included a statement reflecting transmission routes, signs and symptoms associated with COVID-19 was used (Supplementary Table 1). This shortened pathogen disgust survey was based on a pathogen disgust survey previously described by Curtis and de Barra which comprised 75 disgust elicitor statements derived from infectious disease transmission routes (22). To develop our shortened pathogen disgust survey we selected the 30 items which loaded most strongly onto the six factors identified by Curtis and de Barra (22). Invariance testing and cluster analysis were used to determine whether removing the remaining items impacted on the survey structure as described below. Participants were asked to rate their disgust toward each item on a scale from 1–100 from no disgust to extreme disgust. The default position of the scale was set to 50. In addition to these disgust elicitor statements participants were also asked to indicate how often they experienced disgust. Basic demographic data including age, occupation and gender was also collected.

#### Exploratory factor and principal component analysis

Exploratory factor analysis was conducted by splitting the sample into a 2019 cohort (*N* = 299) and 2020 cohort (*N* = 340). Factors that had eigenvalues > 1 (23) were extracted and principal component analysis (PCA) with oblique rotation to extract factors was used, mirroring the analysis conducted by Curtis and de Barra (22). Internal consistency for extracted factors was analyzed using Cronbach's α.

#### Invariance testing

Invariance testing on the pathogen disgust scale was carried out to ensure that measurement of pathogen disgust had not significantly changed before the COVID-19 pandemic (2019 cohort) and during the COVID-19 pandemic (2020 cohort) using the steps described by van de Schoot et al. (24) to test for measurement invariance. Metric invariance was tested by constraining the factor loadings (i.e., how important each question item is to the underlying factor). Finally, scalar invariance which forces both the intercepts and factor loadings to be equal across the 2019 and 2020 cohorts was tested.

### Data collection

All data were collected using the online participant recruitment platform Prolific (www.prolific.co) and the Gorilla Experiment Builder (RRID: SCR_020991) to create and host all experiments. All study participants were aged between 18 and 65 and were UK nationals resident in the UK at the time of the study. The infection status of participants was not determined. Data were collected in two stages; once before the COVID-19 pandemic (09/06/19) (referred to as the 2019 cohort throughout) and once during the pandemic (between 13/03/20 and 07/04/20) (referred to as the 2020 cohort throughout). The 2020 cohort consisted of a repeated sampling of the 2019 cohort (*N* = 151) [referred to as the 2020 cohort (group 1)] and a new sample (*N* = 189) [referred to as the 2020 cohort (group 2)]. All received financial payment for taking part in the study (£1.25).

### Data analysis

#### Regression analysis

Various regression models were conducted, primarily a linear regression and a multilevel linear regression with factor responses (level-1 units) nested within participants (level-2 units). Three models were used to demonstrate that statistically significant improvements in fit could be observed by allowing disgust to vary by participant and that additional assumptions about disgust were supported at each step. The primary aim of the second stage was to establish the effect of COVID-19 on disgust responses while controlling for covariates such as age, gender, and disgust factor.

A series of three multivariate regression analyses were conducted using the following set of predictors: disgust factor, study group, gender, age. In addition to these main effects, several two-way interactions were considered, including study group by disgust factor, gender by disgust factor, age by disgust factor, and gender by age. Finally, two three-way interactions were considered - gender by age by disgust factor and gender by study group by disgust factor. The most important predictors to the current investigation were the main effects of study group (which shows the overall effect that responding during the COVID-19 pandemic had on pathogen disgust), the interaction between study group and disgust factor (which shows the factor specific effect that responding during the COVID-19 pandemic had on pathogen disgust e.g., “Hygiene” disgust or “Animal” disgust).

The first was a linear regression and did not account for the correlation between pathogen disgust responses within each participant (i.e., did not account for the fact that the same participant responded to the six disgust factors).

The second regression analysis used the same set of predictors but accounted for the “nested” structure of our data (with each participant responding to each of the six factors of pathogen disgust) using a random intercept. This allows each participant to have a unique component of their “disgust” response, accounting for individual differences in mean disgust ratings.

The third regression analysis used the same set of predictors as the first and second analysis but introduced a random effect of disgust factor i.e., the influence that a disgust factor (such as hygiene) has on disgust response was allowed to vary between participants. For example, for the majority of participants responding to hygiene factor statements reduced disgust response by 20 points on average compared to lesion factor statements. However, this relationship may not hold for all participants e.g., some participants may find lesion factor statements less disgusting than hygiene statements. The random effects model allows for individual differences in response to each disgust factor.

All analysis was conducted in R version 1.2.5001. Packages used were sjPlot (25), GGally (26), lme4 (RRID: SCR_015654), ggplot2 (RRID: SCR_014601), and psych (RRID: SCR_021744).

### Ethics approval statement

This research has received ethical approval following review by The Open University's Human Research Ethics Committee (HREC/3231/McMullan/Carr) and adheres to all BPS ethics standards. A full information sheet and debrief form were provided and each participant was required to provide written informed consent before being enrolled.

## Results

### Participants

Of the 499 unique participants, 21 either failed to respond correctly to an attention check question or reported an age < 18 or > 65 and were excluded from the analysis. Participants were not excluded on the basis of whether or not they had COVID-19. There were 299 participants in the 2019 cohort and 340 participants in the 2020 cohort. This cohort contained 151 participants who were part of the 2019 cohort (2020 cohort group 1) and 189 new participants (2020 cohort group 2). Participant demographic data is summarized in Table 1.

**Table 1:** Participant demographic data.

	**2019 cohort**	**2020 cohort**			**Whole study (unique participants)**
		**Group 1**	**Group 2**	**Total**	
Size of cohort	299	151	189	340	488
Percentage female	50.8	49.7	47.6	48.8	50.5
Average age (years)	33.48 (s.d. 12.07)	33.76 (s.d. 12.36)	36.5 (s.d. 12.59)	34.98 (s.d. 12.52)	34.3 (s.d. 12.32)
**Employment**	
Professional occupation (undergraduate degree or equivalent required)	47	29	36	65	112
Student	48	18	37	55	103
Administrative and secretarial	50	31	22	53	103
Not currently in work	48	17	28	45	93
Sales and customer service	22	15	17	32	54
Manager or director	22	12	14	26	48
Skilled trade	21	9	14	23	44
Associate professional and technical occupation (high-level vocational qualification or training required)	12	11	15	26	38
Caring or leisure occupation	21	6	5	11	32
Elementary occupation	6	3	1	4	10
Process plant and machine operator	2	-	-	-	2

### Factor analysis and measurement invariance shows that the measurement of pathogen disgust does not vary with COVID-19

Before investigating the effect of the COVID-19 pandemic on pathogen disgust sensitivity we established whether pathogen disgust responses were measured in a similar way before the COVID-19 pandemic and during the COVID-19 pandemic i.e., ensuring that a one point increase in response to a statement represents the same increase in “disgust” for both 2019 and 2020 groups. This stage involved conducting a factor analysis and measurement invariance testing. The first stage had two aims: (1) establish that the underlying factor structure is unchanged between the 2019 and 2020 cohorts and (2) identify whether mean factor scores are comparable between the 2019 and 2020 cohorts.

Similar factors were extracted for both the 2019 and 2020 cohorts (Supplementary Table 2). This factor structure was broadly similar to the factor structure of the 75 item pathogen disgust sensitivity instrument described by Curtis and de Barra that our survey was derived from (22). Therefore, in agreement with Curtis and de Barra (22), we labeled our factors “Hygiene,” “Lesion,” “Food,” “Animal,” “Sex,” and “Atypical” disgust based on the common theme of the statements that loaded onto each of these factors. The factors had broadly the same factor structure with some key differences between the 2019 and 2020 cohorts (Supplementary Table 2). Firstly, some cross-loading was seen in the 2019 cohort. The questionnaire item “Walking in your bare feet, you step on and squash a slug” had a significant loading (>0.3) for both the Animal and Hygiene factors. The loading on Hygiene only marginally passed the definition of a significant loading, 0.304, and was excluded from the Hygiene factor. Secondly, there were some differences in the items that loaded onto the six disgust factors. The items “A hairless old cat rubs up against your leg,” “Eating a sausage 2 weeks past its use by date,” and “Eating onion flavored ice-cream” did not have significant loadings on their respective factors in the 2019 cohort but achieved significant loadings in the 2020 cohort. Cronbach α's for each disgust extracted factors were between 0.73 for the Food factor and 0.88 for the Lesion factor (Hygiene 0.78, Lesion 0.88, Food 0.73, Animal 0.76, Sex 0.83, Atypical 0.79) reflecting satisfactory internal consistency.

Following the exploratory factor analysis, we carried out invariance testing on the pathogen disgust scale to ensure that measurement of pathogen disgust had not significantly changed before the COVID-19 pandemic (2019 cohort) and during the COVID-19 pandemic (2020 cohort). Firstly, to ensure that the underlying factor structure (i.e., what factor each question item relates to) was equivalent between the 2019 and 2020 cohorts we tested for configural invariance. Across a range of criteria [comparative fit index (CFI), Root mean square error approximation **(**RMSEA), and standardized root mean square residual (SRMR)], there was not a significant reduction in model fit when the factor structure was constrained to be equal between the two groups (Table 2) indicating that there was no difference in the underlying factor structure between the two cohorts. Secondly, we tested for metric invariance to determine how important each question item was to the underlying factor. Again, there was not a significant reduction in model fit after constraining the factor loadings to be equal across the 2019 and 2020 (Table 2) implying that there was no difference in the importance of the question between the two cohorts. Finally, we tested for scalar invariance to determine whether that the starting point of the disgust scale was equivalent for both the 2019 and 2020 cohorts i.e., the 2020 cohort might have a higher average disgust response but equivalent measurement across the factor loadings which would upwardly bias the disgust responses taken in 2020 when compared to 2019. Again, we found that there was no reduction in model fit for the model compared to the metric model (Table 2) indicating that measurement in 2019 and 2020 is equivalent allowing for comparison of factor scores.

**Table 2:** Results of invariance testing.

**Model**	**χ2**	**df**	**Comparative fit index (CFI)**	**Root mean square error approximation (RMSEA)**	**Standardized root mean square residual (SRMR)**
Overall model	1,038.823	390	0.904	0.051	0.059
2019 model	668.903	390	0.906	0.049	0.064
2020 model	748.958	390	0.905	0.052	0.064
Configural	1,417.862	780	0.905	0.051	0.064
Metric	1,439.255	804	0.906	0.05	0.065
Scalar	1,474.649	828	0.904	0.049	0.065

Taken together the results of invariance testing indicate that measurement of pathogen disgust in both cohorts was comparable.

### Age, gender and disgust sensitivity

**Figure 1:**
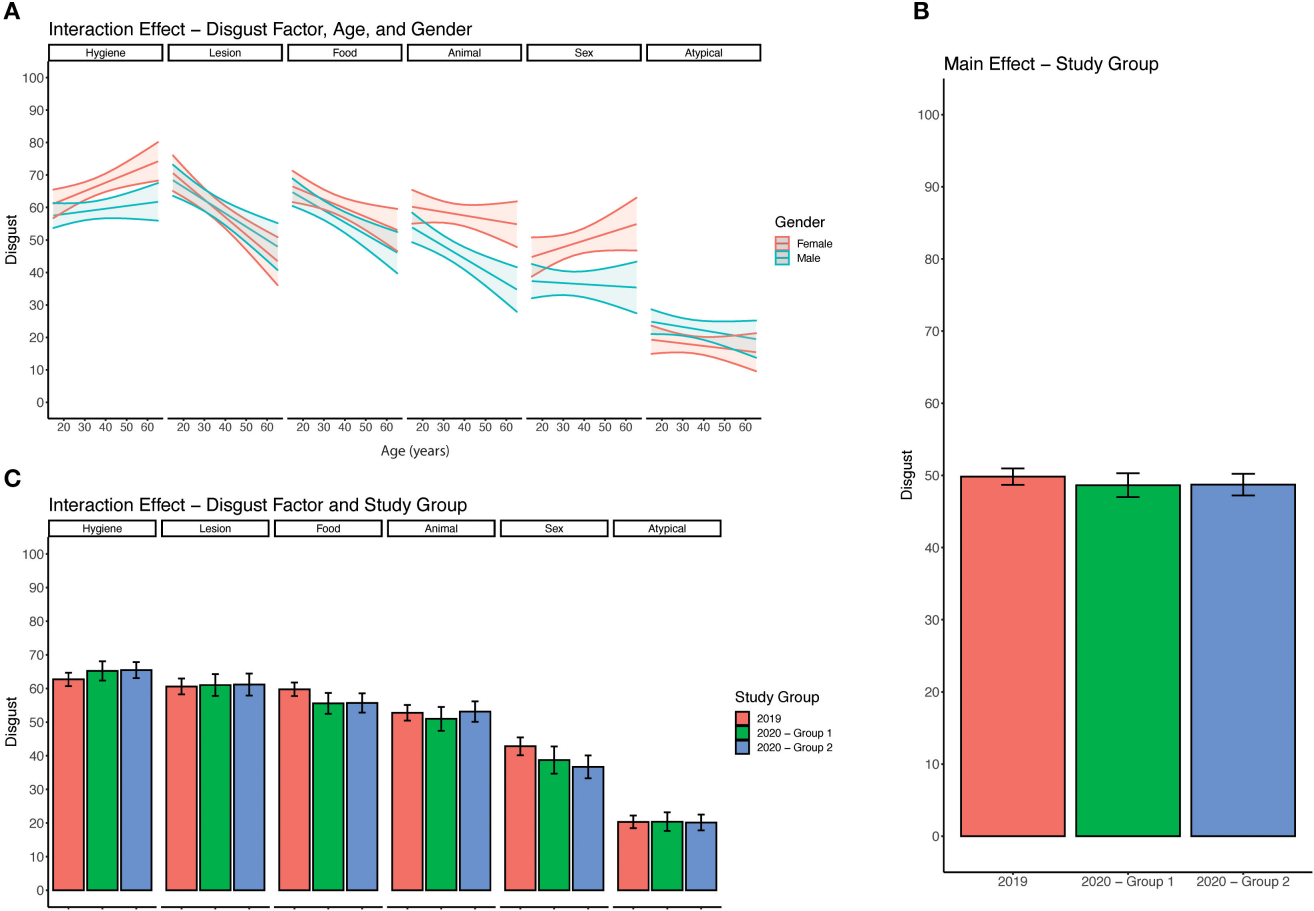
Analysis of disgust responses. **(A)** Three-way interaction between age, gender, and disgust factor. For each disgust factor, a pair of lines with a 95% confidence interval (shaded region) indicates average disgust response across age. **(B)** Average disgust response across all 30 items by study group. 2019 group which refers to the sample collected in 2019. 2020 – Group 1 refers to the subset of responses collected in 2020 that had previously completed the disgust survey. 2020 – Group 2 refers to responses collected in 2020 that had not previously completed the disgust survey. **(C)** Average disgust response across 6 disgust factors separated by study group.

Previous work has demonstrated associations between pathogen disgust sensitivity, age and gender (22). To determine whether there were any interactions between age, gender and pathogen disgust factor in our sample we used a series of multivariate regression analyses as detailed in the Materials and Methods section to consider two-way interactions between gender and pathogen disgust factor and also age and pathogen disgust factor and three-way interactions between age, gender and pathogen disgust factor (Supplementary Table 3 and Figure 1A). Three factors, animal, sex and hygiene disgust, showed a significant three-way interaction with age and gender while no significant interaction was observed between food, lesion or atypical disgust, age and gender. The effect of gender on disgust factors broadly mirrors that observed by Curtis and de Barra who found the most significant effect of gender on sex and animal disgust (22). Given that we observed effects of age and gender on disgust responses we controlled for both of these factors as covariates in our analysis of disgust sensitivity before and during the COVID-19 pandemic.

### COVID-19 and disgust sensitivity

#### The COVID-19 pandemic did not alter overall pathogen disgust responses in this cohort

To determine whether there was an effect of the COVID-19 pandemic on pathogen disgust sensitivity we performed a series of regression analyses comparing pathogen disgust responses in the 2019 and 2020 cohorts as detailed in the Materials and Methods section. We found no significant influence of cohort on pathogen disgust responses (Figure 1B). Both the 2020 cohort (group 1), who had previously completed the survey as part of the 2019 cohort, (β = 1.75, 95% CI: −1.52, 5.02) and 2020 cohort (group 2), who had not previously completed the survey, (β = 2.06, 95% CI: −3.30, 7.42) did not have significantly different pathogen disgust responses compared to the 2019 cohort or each other (Figure 1B).

#### The COVID-19 pandemic did not alter pathogen disgust responses to COVID-19 transmission routes in this cohort

Since overall pathogen disgust sensitivity was not altered during the COVID-19 pandemic (2020 cohort) when compared to baseline pathogen disgust (2019 cohort) we looked at interactions between the 6 disgust factors, identified by our exploratory factor analysis, and our cohorts. In particular, we hypothesized that disgust responses to a statement reflecting COVID-19 disease transmission routes (Supplementary Table 1), which loaded onto the hygiene factor, might be altered during the COVID-19 pandemic. Using regression analysis as detailed in the Materials and Methods section we found a significant interaction between the cohort and one disgust factor (Figure 1C). The 2020 cohort (group 2) had significantly lower disgust responses for the food disgust factor (β = −6.48, 95% CI: −12.36, −0.60) than the 2019 cohort (Figure 1C). No interactions were found between the cohort and other disgust factors including hygiene disgust [2020 cohort (group 2): β = 0.67, 95% CI: −4.99, 6.33; 2020 cohort (group 1): β = 1.03 95% CI: −5.54, 3.48] (Figure 1C).

## Discussion

The pathogen avoidance theory of disgust predicts that disgust sensitivity is associated with the threat of infection. In this study we tested this prediction by comparing pathogen disgust sensitivity during the COVID-19 pandemic in 2020 to baseline pathogen disgust sensitivity in the same sample of UK adults. Given the magnitude of the COVID-19 pandemic, its impact on people's lives and the scale of media coverage relating to the outbreak, we hypothesized that, if disgust sensitivity is correlated with the threat of infection, as predicted by pathogen avoidance theory, individual's disgust sensitivity will be increased during the pandemic when compared to their baseline disgust sensitivity.

### Key findings

To test pathogen disgust sensitivity we utilized an online pathogen disgust survey containing 30 items reflecting signs, symptoms and transmission routes of disease. This instrument was derived from a 75 item pathogen disgust survey previously described by Curtis and de Barra (22). Analysis of our data across both cohorts shows that our shortened version of this survey has a broadly similar factor structure to that previously described by Curtis and de Barra (22), validating their six factor structure model and demonstrating the robustness of the model across multiple cohorts. Furthermore, it demonstrates that a shortened version of the pathogen disgust survey can be used to measure pathogen disgust sensitivity.

Using this shortened pathogen disgust survey we compared overall pathogen disgust sensitivity before and during the 2020 COVID-19 pandemic in a sample of UK adults. When controlling for covariates such as age, gender and disgust factor we found that the COVID-19 pandemic did not alter overall pathogen disgust sensitivity in our sample. Therefore, we accept our null hypothesis that overall pathogen disgust responses are equal during the COVID-19 pandemic and prior to the COVID-19 pandemic.

### Comparisons to existing disgust literature

Our findings are consistent with previous data which do not fully support an association between disgust sensitivity and infection susceptibility or risk of infection (18, 27–29), however they are in contrast to other studies, in different populations under different COVID-19 restrictions, which found that disgust sensitivity was increased during the early stages of the COVID-19 pandemic (19, 20). There are a number of possible explanations why our results may differ from those of past studies on COVID-19 and disgust sensitivity. Firstly, this difference may be due to the disgust sensitivity measures used across studies. In contrast to Milkowska et al. (19) and Stevenson et al. (20), who used measures using disgust elicitor statements based on self-reported lists of disgusting items (16, 21) the pathogen disgust measure in this study uses disgust elicitor statements derived from infectious disease transmission routes (22). Furthermore, the disgust scale (16) and Three Domain disgust scale (21) used by Stevenson et al. (20) and Milkowska et al. (19) are based on a Likert-scale however, in this study pathogen disgust was assessed using a scale from 1–100. These differences may have resulted in differing baseline disgust sensitivity across the measures.

Secondly, our study controlled for co-variates including age and gender therefore demographic differences between studies are unlikely to account for these different findings. However, it should be noted that the proportion of males and females differs across studies. In this study the population was equally balanced with respect to gender in contrast to Stevenson et al. (20) (75% female) and Milkowska et al. (19) (100% female). A supplementary analysis of only the female data from our study gave broadly similar results to our analysis of the whole cohort with the exception of a significant decrease in disgust for the Sex factor in the 2020 (group 2) cohort which was not observed in the analysis of the whole cohort or females in the 2020 (group 1) cohort (data not shown). This data suggests that differences in the proportion of males and females does not account for the different findings of our study.

Thirdly given that each study collected data from a sample taken from a different geographic location, one possible explanation may be that the pandemic altered disgust sensitivity in some countries but not others. Similar patterns of disgust sensitivity have been observed across all regions of the world (14) however differences in the severity of the pandemic and/or the IPC measures introduced in each country may account for the different findings of our study. Related to this point it should be noted that these studies were all conducted at a similar time point prior to the introduction of COVID-19 vaccination programmes and therefore differences in the availability of vaccines between countries do not explain the different findings between studies. Fourthly, since study participants were not asked to report their COVID-19 infection status in any of the three studies it remains possible differences in the prevalence of COVID-19 amongst the different survey cohorts may have served to alter the perceived risk of COVID-19 and therefore disgust sensitivity.

Finally, in contrast to Milkowska et al. (19) and Stevenson et al. (20) who both used matched populations to compare disgust sensitivity pre- and post-pandemic, our study included data collected from a group of individuals whose pathogen disgust sensitivity had been determined prior to the COVID-19 pandemic allowing us to compare responses from the same individuals before and during the COVID-19 pandemic and so reduce the confounding effect of inter-individual differences. Studies investigating differences in disgust sensitivity in response to infection risk in a single population are limited. Previous studies have used natural variation in vulnerability to infection across the female menstrual cycle and during pregnancy to relate disgust sensitivity to physiological changes in vulnerability to infection within individuals (28–31). However, to our knowledge, this is the first study of pathogen disgust sensitivity that collected data from the same group of participants before and during an outbreak of an infectious disease in order to investigate the effect of external changes in the threat of infection.

### The relationship between COVID-19 and pathogen disgust factors

While a general pathogen avoidance response may be appropriate for all infectious disease cues, specific behavioral responses to infection are likely to be related to the nature of the pathogen threat. Therefore, we hypothesized that disgust sensitivity would be greatest toward disgust elicitors that reflect transmission routes, signs and symptoms associated with COVID-19. Our pathogen disgust survey included one statement reflecting COVID-19 transmission routes “Feeling someone cough into your face” and one statement reflecting failure to comply with IPC social distancing measures “On the subway, you are forced to stand close to someone with body odor and greasy hair.” Consistent with previous results (22) both of these statements loaded onto the hygiene disgust factor in both cohorts however when controlling for other covariates we did not find a significant difference in hygiene disgust during the COVID-19 pandemic when compared to baseline responses. Therefore, we accept our null hypothesis that disgust responses to COVID-19 related statements are not significantly altered during the pandemic with the following caveats. Firstly, scores for statements that loaded onto hygiene disgust were relatively high in the 2019 cohort raising the possibility that baseline hygiene disgust was too high to detect any increase as a result of COVID-19. Secondly, our survey was designed prior to the current COVID-19 pandemic and therefore statements were not specifically designed to reflect COVID-19 signs, symptoms, transmission routes or IPC measures. It is possible that the inclusion of further statements reflecting specific aspects of COVID-19 may have revealed effects on specific behavioral responses.

Although COVID-19 did not alter hygiene, lesion, sex, atypical or animal disgust sensitivity we did observe a small but significant decrease in food disgust during the COVID-19 pandemic when compared to baseline. Lowered food disgust does not immediately seem consistent with the hypothesis that disgust responses reflect the nature of the pathogen threat however food disgust sensitivity has been shown to affect eating and food behavior including a positive association between food disgust sensitivity and frequency of wasting food (32). Interestingly food disgust sensitivity has been identified as a predictor of shopping behavior and disease preventative behavior related to the COVID-19 pandemic with higher food disgust associated with shopping behavior aimed at reducing exposure to the virus such as purchasing pre-packed and long-life foods (33). Our study did not specifically address individual's eating and food behavior however changes in shopping and eating habits such as a more relaxed attitude to best before dates and a reduction in food waste in the early stages of lockdown (when data from cohort 2 was collected) have been reported (34, 35) raising the possibility that the lowered food disgust that we observed could be associated with changes in shopping and eating habits during the pandemic.

Although our findings do not support an association between overall disgust sensitivity or hygiene disgust and threat of infection by COVID-19, they do reveal possible associations with other disgust factors that it is tempting to speculate could be attributed to consequences of IPC measures.

### Study strengths and limitations

Previous studies exploring the relationship between COVID-19 and disgust sensitivity have used a between-participant design in which data was collected from different cohorts of individuals pre- and post-pandemic which does not allow disgust sensitivity in the same group of individuals to be compared pre- and post-pandemic. Thus, despite cohorts being matched, it remains possible that the observed differences in disgust sensitivity are a result of variation between individuals in the cohorts rather than as a result of increased threat of infection during the pandemic. The main strength of this study is the use of both within- and matched-subjects approaches which find replicable results. However, while our data may provide further insight into the relationship between COVID-19 and disgust sensitivity which can be used to inform the design of public health messages to promote uniform behavior change they are not without their limitations. Firstly, our survey was designed prior to the current COVID-19 pandemic and therefore statements were not specifically designed to reflect COVID-19 signs, symptoms, transmission routes or IPC measures. It is possible that the inclusion of further statements reflecting specific aspects of COVID-19 may have revealed effects on specific behavioral responses. Related to this point our findings do not exclude the possibility that epidemic or pandemic diseases with different signs, symptoms or transmission routes could alter individual's disgust sensitivity. Further studies comparing baseline pathogen disgust sensitivity to disgust sensitivity during other disease outbreaks with varying transmission routes, signs and symptoms in the same group of individuals are needed to determine whether pathogen disgust sensitivity is associated with threat of infection in other contexts. Secondly, as previously mentioned the COVID-19 status of participants or their experience of the pandemic were not determined in our study. Factors such as recent or current infection with COVID-19, hospitalization or death of a family member, inclusion in a vulnerable/at risk group and the degree to which they were involved in employment that increased their exposure to COVID-19 may all have served to alter participants perceived risk of COVID-19 and therefore their disgust sensitivity.

### Conclusion and implications

Understanding the psychological, behavioral and cultural factors that influence compliance with evidence-based IPC measures such as social distancing can help to inform the design of public health messages to promote more uniform behavioral change. Disgust sensitivity appears to be an important predictor of individual's responses to the COVID-19 pandemic (7–10) leading to suggestions that emphasizing aspects of the virus that induce feelings of disgust could be used to promote behavioral change and improve compliance with public health measures designed to tackle COVID-19. Disgust has previously been leveraged in this way to influence social behaviors such as hand washing to prevent the spread of disease. For example, during the 2009/10 H1N1 influenza pandemic, the UK Government's information leaflet which was delivered to every house in the UK depicted the aerosol spread of a sneeze on its cover. Exposure to this material was associated with increases in hygienic behavior although disgust was not explicitly evaluated (36). Our study suggests that current IPC measures, public health messaging, media coverage and other factors associated with the 2020 COVID-19 pandemic do not alter people's overall disgust sensitivity or their disgust in relation to symptoms, signs and transmission routes for COVID-19. Indeed, evidence shows people tend to show solidarity and cooperation in times of emergency (37) and UK Government public health messaging during the COVID-19 lockdown promoted social responsibility and moral values associated with caring for others. However, our findings do not exclude the possibility that novel interventions targeting disgust could be leveraged to promote compliance with IPC related to COVID-19.

## Data availability statement

The datasets presented in this study can be found in online repositories. The names of the repository/repositories and accession number (s) can be found at: Open Research Data Online (ORDO) repository https://doi.org/10.21954/ou.rd.13109831.v1.

## Ethics statement

The studies involving human participants were reviewed and approved by the Open University Human Research Ethics Committee. The patients/participants provided their written informed consent to participate in this study.

## Author contributions

CJH and RM designed the study. PC and EB collected the data. PC analyzed the data. All authors interpreted the data, prepared the manuscript, and approved the final version before submission.

## Funding

This work was supported by funding from The Open University Health and Wellbeing Priority Research Area to RM and CJH (https://www.open.ac.uk/health-wellbeing/). The funders had no role in study design, data collection and analysis, decision to publish, or preparation of the manuscript.

## Conflict of interest

The authors declare that the research was conducted in the absence of any commercial or financial relationships that could be construed as a potential conflict of interest.

## Publisher's note

All claims expressed in this article are solely those of the authors and do not necessarily represent those of their affiliated organizations, or those of the publisher, the editors and the reviewers. Any product that may be evaluated in this article, or claim that may be made by its manufacturer, is not guaranteed or endorsed by the publisher.
