# Supplementary material for: The effect of the COVID-19 pandemic on disgust sensitivity in a sample of UK adults
Source: Front Public Health. 2022 Oct 27;10:1020850. doi: 10.3389/fpubh.2022.1020850 (PMC9648408; doi:10.3389/fpubh.2022.1020850)
Supplement: Supplementary file 1 [file Data_Sheet_1.docx]

**Supplemental Table 1.** **Pathogen disgust survey.**

| Listening to someone sniffle and snort continually |
| --- |
| Seeing some snotty tissues left on the table |
| Watching a woman pick her nose |
| Feeling someone cough into your face |
| On the subway, you are forced to stand close to someone with body odour and greasy hair |
| Helping a friend cook dinner you have to take the innards out of a raw chicken |
| Walking in your bare feet, you step on and squash a slug |
| After losing a bet, you have to hold a fat wriggling worm in your bare hands for 60s |
| You are served a dish made of cow's tongue and cheek |
| On television you see someone eat a raw fish head |
| Hearing about a woman who had sex with seven people in one day |
| A street prostitute offers you sex for money |
| Shortly after meeting someone, you take them back to your house and have sex |
| You discover that your romantic partner once paid for sexual intercourse |
| A friend admits to attempting sexual intercourse with a piece of fruit |
| Sharing an elevator with a man with a disfigured face |
| Shaking hands with a homeless man |
| Shaking hands with someone missing a thumb |
| In a crowd you notice a man with one empty eye socket |
| A hairless old cat rubs up against your leg |
| On a medical TV programme you see some blisters on a male's genitals |
| Seeing pus come from a genital sore |
| You see a nurse dressing an infected wound; under the yellow bandages there is a weeping sore |
| Your friend shows you a big, oozing lesion on his foot |
| Someone you work with develops a bad eye infection; the eye is almost fully sealed and weeps constantly |
| You pour lumpy stale milk on your cereal |
| Finding a furry green patch on a loaf of bread |
| Eating a sausage two weeks past its use by date |
| Biting into soft, brown bruise on an apple |
| Eating onion flavoured ice-cream |

Statements reflecting non-compliance with IPC control measures, transmission routes, signs and symptoms associated with COVID-19 are highlighted.

**Supplemental Table 2. Pattern matrix for factor analysis.**

| Oblimin Rotation - Six Factors | **2019** | | | | | | **2020** | | | | | | **Whole Sample** | | | | | |
| --- | --- | --- | --- | --- | --- | --- | --- | --- | --- | --- | --- | --- | --- | --- | --- | --- | --- | --- |
|  | **Lesion** | **Atypical** | **Sex** | **Hygiene** | **Animal** | **Food** | **Lesion** | **Atypical** | **Sex** | **Hygiene** | **Animal** | **Food** | **Lesion** | **Atypical** | **Sex** | **Hygiene** | **Animal** | **Food** |
| NorSnif | - | - | - | 0.676 | - | - | - | - | - | 0.717 | - | - | - | - | - | 0.713 | - | - |
| BWSnot | - | - | - | 0.671 | - | - | - | - | - | 0.769 | - | - | - | - | - | 0.714 | - | - |
| NorNose | - | - | - | 0.628 | - | - | - | - | - | 0.608 | - | - | - | - | - | 0.620 | - | - |
| DrpCough | - | - | - | 0.569 | - | - | - | - | - | 0.570 | - | - | - | - | - | 0.585 | - | - |
| ContOdour | - | - | - | 0.584 | - | - | - | - | - | 0.467 | - | - | - | - | - | 0.535 | - | - |
| FURaw | - | - | - | - | 0.638 | - | - | - | - | - | 0.763 | - | - | - | - | - | 0.715 | - |
| AniSlug | - | - | - | 0.304 | 0.410 | - | - | - | - |  | 0.417 | - | - | - | - | - | 0.406 | - |
| AniWorm | - | - | - | - | 0.425 | - | - | - | - | - | 0.538 | - | - | - | - | - | 0.487 | - |
| FUCow | - | - | - | - | 0.667 | - | - | - | - | - | 0.631 | - | - | - | - | - | 0.653 | - |
| FUFish | - | - | - | - | 0.570 | - | - | - | - | - | 0.614 | - | - | - | - | - | 0.581 | - |
| SexSeven | - | - | 0.680 | - | - | - | - | - | 0.821 | - | - | - | - | - | 0.766 | - | - | - |
| SexProst | - | - | 0.629 | - | - | - | - | - | 0.768 | - | - | - | - | - | 0.705 | - | - | - |
| SexShort | - | - | 0.735 | - | - | - | - | - | 0.697 | - | - | - | - | - | 0.710 | - | - | - |
| SexPaid | - | - | 0.695 | - | - | - | - | - | 0.695 | - | - | - | - | - | 0.701 | - | - | - |
| NorFruit | - | - | 0.460 | - | - | - | - | - | 0.472 | - | - | - | - | - | 0.478 | - | - | - |
| BSFace | - | 0.820 | - | - | - | - | - | 0.843 | - | - | - | - | - | 0.841 | - | - | - | - |
| ConHome | - | 0.568 | - | - | - | - | - | 0.473 | - | - | - | - | - | 0.512 | - | - | - | - |
| BSThumb | - | 0.842 | - | - | - | - | - | 0.738 | - | - | - | - | - | 0.786 | - | - | - | - |
| BSEye | - | 0.681 | - | - | - | - | - | 0.725 | - | - | - | - | - | 0.689 | - | - | - | - |
| AmiCat | - | - | - | - | - | - | - | 0.504 | - | - | - | - | - | 0.382 | - | - | - | - |
| SexBlis | 0.656 | - | - | - | - | - | 0.691 | - | - | - | - | - | 0.677 | - | - | - | - | - |
| SexPus | 0.801 | - | - | - | - | - | 0.757 | - | - | - | - | - | 0.786 | - | - | - | - | - |
| DisWound | 0.773 | - | - | - | - | - | 0.826 | - | - | - | - | - | 0.795 | - | - | - | - | - |
| DisLesio | 0.830 | - | - | - | - | - | 0.814 | - | - | - | - | - | 0.826 | - | - | - | - | - |
| DisEye | 0.630 | - | - | - | - | - | 0.634 | - | - | - | - | - | 0.632 | - | - | - | - | - |
| FSMilk | - | - | - | - | - | 0.605 | - | - | - | - | - | 0.658 | - | - | - | - | - | 0.676 |
| FSBread | - | - | - | - | - | 0.594 | - | - | - | - | - | 0.494 | - | - | - | - | - | 0.553 |
| FSSaus | - | - | - | - | - | - | - | - | - | - | - | 0.401 | - | - | - | - | - | 0.332 |
| FSApple | - | - | - | - | - | 0.622 | - | - | - | - | - | 0.563 | - | - | - | - | - | 0.597 |
| FUIce | - | - | - | - | - | - | - | - | - | - | - | 0.466 | - | - | - | - | - | 0.399 |
| **Variance by Factor** | **0.096** | **0.083** | **0.075** | **0.073** | **0.057** | **0.047** | **0.096** | **0.084** | **0.086** | **0.072** | **0.067** | **0.054** | **0.096** | **0.084** | **0.086** | **0.072** | **0.067** | **0.054** |

**Supplemental Table 3. Regression analyses.**

|  | **Linear Model** | | | | **Linear Model - Random Intercept** | | | | **Mixed Effects - Random Factor Effect** | | | |
| --- | --- | --- | --- | --- | --- | --- | --- | --- | --- | --- | --- | --- |
| *Predictors* | *Estimates* | *CI* | | *p* | *Estimates* | *CI* | | *p* | *Estimates* | *CI* | | *p* |
| Intercept | 77.87 | 70.80 | 84.94 | **<0.001** | 78.98 | 71.63 | 86.32 | **<0.001** | 78.67 | 70.30 | 87.04 | **<0.001** |
| Hygiene | -22.40 | -32.40 | -12.39 | **<0.001** | -22.40 | -30.82 | -13.97 | **<0.001** | -21.47 | -30.15 | -12.80 | **<0.001** |
| Animal | -16.52 | -26.52 | -6.52 | **0.001** | -16.52 | -24.94 | -8.10 | **<0.001** | -16.86 | -27.24 | -6.47 | **0.001** |
| Sex | -38.86 | -48.86 | -28.86 | **<0.001** | -38.86 | -47.29 | -30.44 | **<0.001** | -36.94 | -48.18 | -25.70 | **<0.001** |
| Atypical | -57.83 | -67.83 | -47.83 | **<0.001** | -57.83 | -66.25 | -49.41 | **<0.001** | -58.25 | -66.89 | -49.62 | **<0.001** |
| Food | -7.95 | -17.95 | 2.05 | 0.12 | -7.95 | -16.37 | 0.47 | 0.06 | -8.18 | -17.23 | 0.86 | 0.08 |
| New 2020 | 2.36 | -2.63 | 7.35 | 0.35 | 2.19 | -2.78 | 7.17 | 0.39 | 2.06 | -3.30 | 7.42 | 0.45 |
| Repeated 2020 | 2.43 | -2.99 | 7.85 | 0.38 | 2.83 | -1.83 | 7.49 | 0.23 | 1.75 | -1.52 | 5.02 | 0.30 |
| Gender (Male) | -3.67 | -13.06 | 5.71 | 0.44 | -5.01 | -14.73 | 4.70 | 0.31 | -4.18 | -15.19 | 6.84 | 0.46 |
| Age | -0.53 | -0.71 | -0.34 | **<0.001** | -0.56 | -0.75 | -0.36 | **<0.001** | -0.54 | -0.76 | -0.32 | **<0.001** |
| Gender (Male) * Age | 0.14 | -0.11 | 0.38 | 0.28 | 0.17 | -0.09 | 0.43 | 0.20 | 0.13 | -0.16 | 0.43 | 0.37 |
| New 2020 * Gender | -3.72 | -10.79 | 3.35 | 0.30 | -3.49 | -10.53 | 3.56 | 0.33 | -3.09 | -10.67 | 4.48 | 0.42 |
| Repeated 2020 * Gender (Male) | -1.38 | -8.97 | 6.21 | 0.72 | -0.59 | -7.11 | 5.93 | 0.86 | 0.83 | -3.72 | 5.39 | 0.72 |
| Hygiene * New 2020 | 0.67 | -6.39 | 7.72 | 0.85 | 0.67 | -5.27 | 6.61 | 0.83 | 0.67 | -4.99 | 6.33 | 0.82 |
| Animal * New 2020 | 0.99 | -6.07 | 8.04 | 0.78 | 0.99 | -4.95 | 6.93 | 0.74 | 1.02 | -5.67 | 7.70 | 0.77 |
| Sex * New 2020 | -6.77 | -13.82 | 0.29 | 0.06 | -6.77 | -12.71 | -0.83 | **0.03** | -6.88 | -14.09 | 0.32 | 0.06 |
| Atypical * New 2020 | 0.92 | -6.13 | 7.98 | 0.80 | 0.92 | -5.02 | 6.86 | 0.76 | 1.02 | -4.61 | 6.66 | 0.72 |
| Food * New 2020 | -6.53 | -13.59 | 0.52 | 0.07 | -6.53 | -12.47 | -0.59 | **0.03** | -6.48 | -12.36 | -0.60 | **0.03** |
| Hygiene * Repeated 2020 | -2.58 | -10.25 | 5.09 | 0.51 | -2.58 | -9.04 | 3.88 | 0.43 | -1.03 | -5.54 | 3.48 | 0.66 |
| Animal *Repeated 2020 | -2.18 | -9.85 | 5.49 | 0.58 | -2.18 | -8.64 | 4.28 | 0.51 | -1.39 | -6.01 | 3.23 | 0.56 |
| Sex *Repeated 2020 | -4.61 | -12.28 | 3.06 | 0.24 | -4.61 | -11.07 | 1.85 | 0.16 | -2.25 | -6.91 | 2.40 | 0.34 |
| Atypical * Repeated 2020 | -0.73 | -8.39 | 6.94 | 0.85 | -0.73 | -7.18 | 5.73 | 0.83 | 0.26 | -4.24 | 4.76 | 0.91 |
| Food * Repeated 2020 | -4.70 | -12.36 | 2.97 | 0.23 | -4.70 | -11.15 | 1.76 | 0.15 | -3.54 | -8.07 | 0.99 | 0.13 |
| Hygiene * Gender (Male) | 4.93 | -8.34 | 18.20 | 0.47 | 4.93 | -6.24 | 16.11 | 0.39 | 3.25 | -8.20 | 14.70 | 0.58 |
| Animal * Gender (Male) | 1.37 | -11.90 | 14.64 | 0.84 | 1.37 | -9.80 | 12.55 | 0.81 | 1.93 | -11.77 | 15.63 | 0.78 |
| Sex * Gender (Male) | 3.87 | -9.40 | 17.14 | 0.57 | 3.87 | -7.30 | 15.05 | 0.50 | 0.35 | -14.45 | 15.16 | 0.96 |
| Atypical * Gender (Male) | 10.47 | -2.80 | 23.74 | 0.12 | 10.47 | -0.70 | 21.65 | 0.07 | 10.18 | -1.22 | 21.57 | 0.08 |
| Food * Gender (Male) | 3.63 | -9.65 | 16.90 | 0.59 | 3.63 | -7.55 | 14.80 | 0.53 | 3.95 | -7.99 | 15.88 | 0.52 |
| Hygiene * Age | 0.83 | 0.57 | 1.09 | **<0.001** | 0.83 | 0.61 | 1.05 | **<0.001** | 0.80 | 0.57 | 1.03 | **<0.001** |
| Animal * Age | 0.42 | 0.16 | 0.68 | **0.001** | 0.42 | 0.21 | 0.64 | **<0.001** | 0.43 | 0.16 | 0.71 | **0.002** |
| Sex * Age | 0.80 | 0.54 | 1.06 | **<0.001** | 0.80 | 0.58 | 1.02 | **<0.001** | 0.74 | 0.45 | 1.04 | **<0.001** |
| Atypical * Age | 0.45 | 0.20 | 0.71 | **0.00** | 0.45 | 0.24 | 0.67 | **<0.001** | 0.46 | 0.24 | 0.69 | **<0.001** |
| Food * Age | 0.27 | 0.01 | 0.53 | **0.04** | 0.27 | 0.05 | 0.49 | **0.02** | 0.27 | 0.04 | 0.51 | **0.03** |
| Hygiene * Gender (Male) * Age | -0.37 | -0.72 | -0.02 | **0.04** | -0.37 | -0.66 | -0.07 | **0.02** | -0.31 | -0.62 | -0.01 | **0.05** |
| Animal* Gender (Male) * Age | -0.39 | -0.74 | -0.04 | **0.03** | -0.39 | -0.69 | -0.10 | **0.01** | -0.41 | -0.77 | -0.04 | **0.03** |
| Sex *Gender (Male) * Age | -0.48 | -0.83 | -0.13 | **0.01** | -0.48 | -0.78 | -0.19 | **0.00** | -0.37 | -0.77 | 0.02 | 0.06 |
| Atypical * Gender (Male) * Age | -0.18 | -0.53 | 0.17 | 0.32 | -0.18 | -0.47 | 0.12 | 0.24 | -0.16 | -0.47 | 0.14 | 0.29 |
| Food * Gender (Male) * Age | -0.23 | -0.58 | 0.12 | 0.20 | -0.23 | -0.52 | 0.06 | 0.13 | -0.24 | -0.56 | 0.08 | 0.15 |
| Hygiene* New 2020 * Gender (Male) | 3.97 | -6.02 | 13.97 | 0.44 | 3.97 | -4.44 | 12.39 | 0.36 | 3.79 | -4.22 | 11.80 | 0.35 |
| Animal * New 2020 * Gender (Male) | -1.04 | -11.04 | 8.96 | 0.84 | -1.04 | -9.46 | 7.38 | 0.81 | -1.04 | -10.50 | 8.42 | 0.83 |
| Sex *New 2020 * Gender (Male) | 1.64 | -8.36 | 11.64 | 0.75 | 1.64 | -6.78 | 10.06 | 0.70 | 1.53 | -8.66 | 11.72 | 0.77 |
| Atypical * New 2020 * Gender (Male) | -3.09 | -13.09 | 6.90 | 0.54 | -3.09 | -11.51 | 5.32 | 0.47 | -3.36 | -11.33 | 4.62 | 0.41 |
| Food *New 2020 *Gender (Male) | 4.43 | -5.57 | 14.43 | 0.39 | 4.43 | -3.99 | 12.85 | 0.30 | 4.34 | -3.98 | 12.66 | 0.31 |
| Hygiene* Repeated 2020 *Gender (Male) | 6.04 | -4.70 | 16.78 | 0.27 | 6.04 | -3.00 | 15.08 | 0.19 | 3.36 | -2.92 | 9.64 | 0.29 |
| Animal * Repeated 2020 * Gender (Male) | -0.30 | -11.04 | 10.43 | 0.96 | -0.30 | -9.35 | 8.74 | 0.95 | -0.18 | -6.61 | 6.25 | 0.96 |
| Sex * Repeated 2020 *Gender (Male) | -1.77 | -12.50 | 8.97 | 0.75 | -1.77 | -10.81 | 7.28 | 0.70 | -3.51 | -9.99 | 2.96 | 0.29 |
| Atypical * New 2020 * Gender (Male) | -1.50 | -12.24 | 9.23 | 0.78 | -1.50 | -10.55 | 7.54 | 0.75 | -3.17 | -9.44 | 3.11 | 0.32 |
| Food * Repeated 2020 * Gender (Male) | -0.26 | -11.00 | 10.47 | 0.96 | -0.26 | -9.31 | 8.78 | 0.95 | -1.10 | -7.41 | 5.22 | 0.73 |
| **Random Effects** |  | | | | | | | | | | | |
| σ^2^ |  | | | | 263.81 | | | | 109.27 | | | |
| τ_00_ |  | | | | 106.03 _ID_ | | | | 320.27 _ID_ | | | |
| τ_11_ |  | | | |  | | | | 259.43 _ID.disgust_factorHygiene_ | | | |
|  |  | | | |  | | | | 448.87 _ID.disgust_factorAnimal_ | | | |
|  |  | | | |  | | | | 556.65 _ID.disgust_factorSex_ | | | |
|  |  | | | |  | | | | 255.41 _ID.disgust_factorAtypical_ | | | |
|  |  | | | |  | | | | 297.71 _ID.disgust_factorFood_ | | | |
| ρ_01_ |  | | | |  | | | | -0.71 | | | |
|  |  | | | |  | | | | -0.65 | | | |
|  |  | | | |  | | | | -0.56 | | | |
|  |  | | | |  | | | | -0.72 | | | |
|  |  | | | |  | | | | -0.63 | | | |
| ICC |  | | | | 0.29 | | | | 0.71 | | | |
| N |  | | | | 488 _ID_ | | | | 488 _ID_ | | | |
| Observations | 3834 | | | | 3834 | | | | 3834 | | | |
| R^2^ / R^2^ adjusted | 0.413 / 0.405 | | | | 0.411 / 0.580 | | | | 0.410 / 0.826 | | | |
